# Diagnostic accuracy of frozen section biopsy for early gastric cancer extent during endoscopic submucosal dissection: a prospective study

**DOI:** 10.1007/s00464-023-10100-2

**Published:** 2023-05-22

**Authors:** Mayu Kobashi, Shigenao Ishikawa, Tomoki Inaba, Masaya Iwamuro, Yuki Aoyama, Tomo Kagawa, Yasuto Takeuchi, Midori Ando, Satoko Nakamura, Hiroyuki Okada

**Affiliations:** 1 Department of Gastroenterology, Kagawa Prefectural Central Hospital, 1-2-1 Asahi-machi, Takamatsu, Kagawa 760-8557 Japan; 2 Department of Gastroenterology and Hepatology, Okayama University Graduate School of Medicine, Dentistry, and Pharmaceutical Sciences, 2-5-1 Shikata-cho, Kita-ku, Okayama, 700-8558 Japan; 3 Department of Regenerative Medicine, Center for Innovative Clinical Medicine, Okayama University Hospital, 2-5-1 Shikata-cho, Kita-ku, Okayama, 700-8558 Japan; 4 Department of Pathology, Kagawa Prefectural Central Hospital, 1-2-1 Asahi-machi, Takamatsu, Kagawa 760-8557 Japan

**Keywords:** Frozen section, Pathological diagnosis, Diagnostic accuracy, Early gastric cancer, Endoscopic submucosal dissection, Lateral margin

## Abstract

**Background:**

Accurate diagnosis of the lateral extent of early gastric cancer during endoscopic submucosal dissection (ESD) is crucial to achieve negative resection margins. Similar to intraoperative consultation with a frozen section in surgery, rapid frozen section diagnosis with endoscopic forceps biopsy may be useful in assessing tumor margins during ESD. This study aimed to evaluate the diagnostic accuracy of frozen section biopsy.

**Methods:**

We prospectively enrolled 32 patients undergoing ESD for early gastric cancer. Biopsy samples for the frozen sections were randomly collected from fresh resected ESD specimens before formalin fixation. Two different pathologists independently diagnosed 130 frozen sections as “neoplasia,” “negative for neoplasia,” or “indefinite for neoplasia,” and the frozen section diagnosis was compared with the final pathological results of the ESD specimens.

**Results:**

Among the 130 frozen sections, 35 were from cancerous areas, and 95 were from non-cancerous areas. The diagnostic accuracies of the frozen section biopsies by the two pathologists were 98.5 and 94.6%, respectively. Cohen’s kappa coefficient of diagnoses by the two pathologists was 0.851 (95% confidence interval: 0.837–0.864). Incorrect diagnoses resulted from freezing artifacts, a small volume of tissue, inflammation, the presence of well-differentiated adenocarcinoma with mild nuclear atypia, and/or tissue damage during ESD.

**Conclusions:**

Pathological diagnosis of frozen section biopsy is reliable and can be applied as a rapid frozen section diagnosis for evaluating the lateral margins of early gastric cancer during ESD.

**Supplementary Information:**

The online version contains supplementary material available at 10.1007/s00464-023-10100-2.

The development of the endoscopic submucosal dissection (ESD) procedure has enabled minimally invasive and curative treatment for early gastric cancer, and the technique has been widely accepted as a standard treatment [1–3]. Although the endoscopic diagnosis of early gastric cancer extent has recently improved by chromoendoscopy using indigo carmine dye spraying [4] and magnifying endoscopy with narrow band imaging [5–7], the overall incidence of positive lateral margins in en bloc specimens of gastric cancers resected by ESD has been reported to be 2.0–6.3% [8–10]; 1.0–6.3% in differentiated [11–13] and 27.3% in undifferentiated cancers [14]. Positive lateral margins in an ESD specimen, as well as in segmental resections during ESD, are known to be a risk factor for local recurrence [8, 15]. Therefore, accurate diagnosis of the lateral extent of lesions is important to achieve en bloc and negative resection margins [16, 17].

Previous studies reveal that the predictive factors for inaccurate determination of the lateral extent of early gastric cancer indicated for ESD include the following: cancer developed after eradication therapy of *Helicobacter pylori* (*H. pylori*), location in the upper third of the stomach, flat type cancer, and undifferentiated-type cancer[8, 11, 18–21]. Marking methods for circumferential biopsies to confirm non-neoplastic mucosa in preoperative esophagogastroduodenoscopy [22–24] help determine the lateral extent of the lesion, but biopsy scars are often undetectable during ESD.

During surgical resection of gastric cancer, microscopic margin analysis using a frozen section has been commonly performed to evaluate proximal and distal resection margins, and conversion from an R1 to an R0 resection, because intraoperative consultation (IOC) on surgical margins is associated with a decreased rate of local recurrence [25, 26]. Therefore, we hypothesized that, during gastric ESD, rapid frozen section analysis using endoscopic biopsy samples to assess margins might be useful, especially for lesions with unclear extents. However, the diagnostic accuracy of frozen section biopsy for gastric cancer has not been investigated. The purpose of this study was to clarify the diagnostic accuracy of the frozen section from gastric biopsy samples in anticipation of rapid frozen section diagnosis during gastric ESD in clinical practice.

## Materials and methods

### Patients and classification of lesions

We prospectively enrolled 32 patients undergoing ESD for early gastric cancer from January 1 to November 31, 2019, at Kagawa Prefectural Central Hospital. We defined the macroscopic types of tumors as elevated (0-I and 0-IIa), flat (0-IIb), and depressed (0-IIc) types according to the Paris endoscopic classification [27]. The location of tumors was classified into the upper, middle, and lower thirds of the stomach. In all patients, gastric lesions had been pathologically evaluated using endoscopic forceps biopsy (Olympus Co Tokyo Japan, FB-25 K-1) and a histologic diagnosis of gastric adenocarcinoma was confirmed prior to enrollment. Gastric cancer was classified into the following five histologic types according to the Japanese classification of Gastric Cancer [28], which is consistent with the WHO classification [29]; papillary adenocarcinoma (pap), well-differentiated tubular adenocarcinoma (tub1), moderately differentiated tubular adenocarcinoma (tub2), poorly differentiated adenocarcinoma (por), and signet ring cell carcinoma (sig). Based on the pathological diagnosis of biopsies before ESD, we defined histologic types of pap, tub1, and tub2 as differentiated-type cancers, and por and sig as undifferentiated-type cancers. All endoscopic procedures in the present study were performed by an expert endoscopist with over 500 ESD cases of experience.

### Study design

**Fig. 1:**
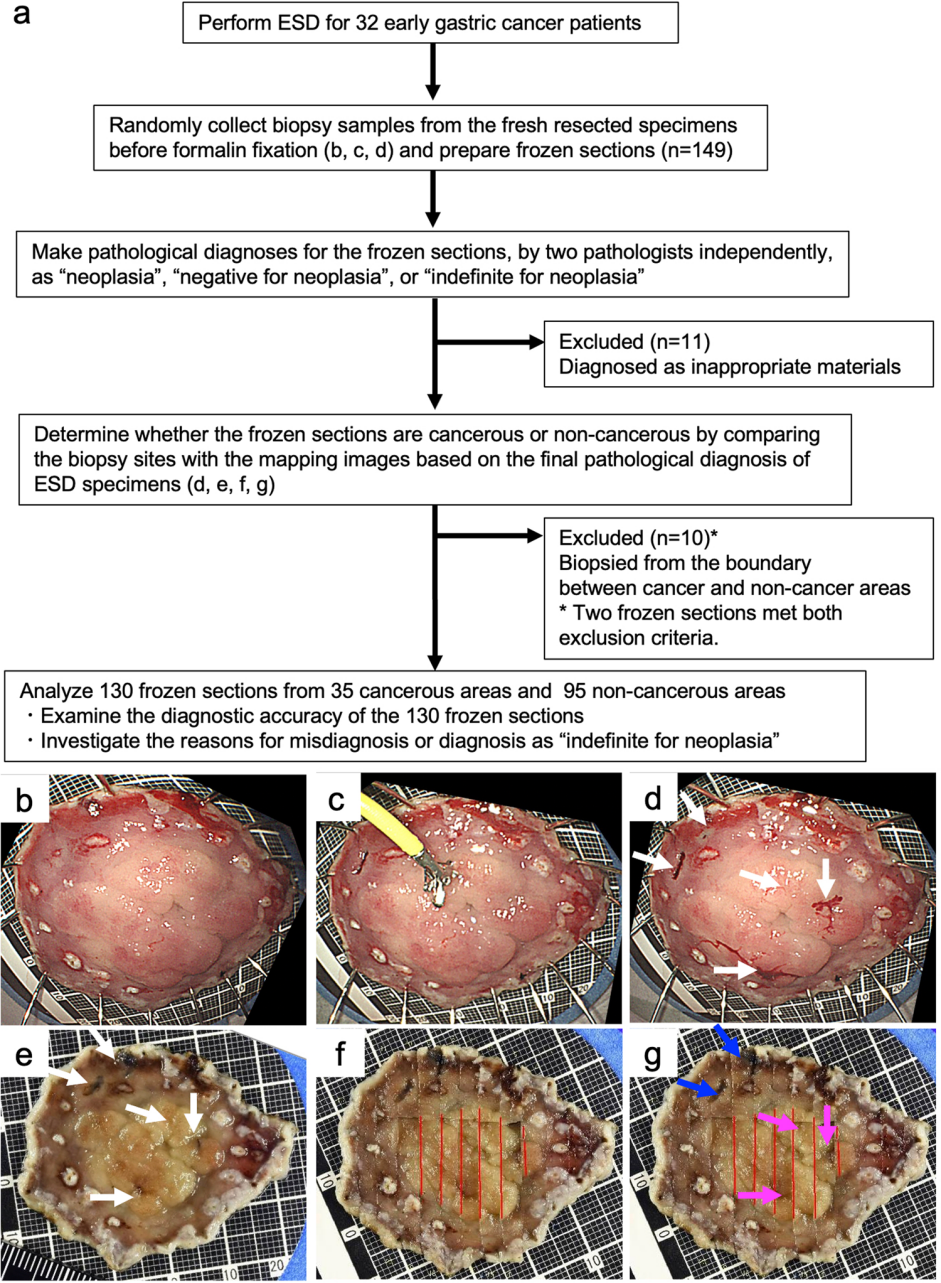
**a** Flowchart of the study. **b** Fresh specimen resected by ESD. **c** Randomly collect biopsy samples. **d** Biopsy sites on the fresh specimen (white arrows). **e** Biopsy sites on the formalin-fixed specimen (white arrows). **f** Mapping image tracing the extent of the lesion (red lines) based on the final pathological diagnosis. **g** Determine whether the frozen section is from the cancerous area (pink arrows) or the non-cancerous area (blue arrows)

The flowchart of this study is shown in Fig. 1a. Immediately after completion of ESD, biopsy samples for the frozen sections were randomly collected from cancerous and non-cancerous areas of the fresh resected ESD specimens before formalin fixation (Fig. 1b, c). The sample acquisition for the frozen sections was also performed by the endoscopist who performed the ESD using endoscopic biopsy forceps. In total, 149 biopsy samples were obtained, and their frozen sections prepared. A pathologist with 20 years of experience (Pathologist A) and another pathologist with 10 years of experience (Pathologist B) participated in this study. Information regarding differentiated- or undifferentiated-type carcinoma in the lesion based on the pathology results of the preoperative biopsy was given to the pathologists before evaluation of the frozen sections. Two pathologists then independently made pathological diagnoses for the 149 frozen sections prepared from the 149 biopsy samples. The pathologists classified the frozen sections into three categories: “neoplasia,” “negative for neoplasia,” and “indefinite for neoplasia.” The diagnosis of “neoplasia” included non-invasive low-grade neoplasia, non-invasive high-grade neoplasia, and invasive neoplasia.

The specimens resected by ESD were fixed with formalin solution after sampling for frozen sections and provided for histopathological diagnosis. Histopathological examination of ESD specimens was made according to the Japanese classification of Gastric Cancer [28] and defined as the gold standard for this study. When multiple histological types coexisted in a lesion, the type with the largest relative area was adopted as the final pathological diagnosis.

Macroscopic photographs of the ESD specimens were taken to identify the sites where biopsies were taken for frozen sections (Fig. 1d). Parallel incisions on the ESD specimens were made at intervals of 2–3 mm and macroscopic photographs were taken again to reconstruct the extent (mapping) of the tumors (Fig. 1e, f) [24]. To determine whether a frozen section was cancerous or non-cancerous, we compared the biopsy sites for the frozen section with the mapping image of the ESD specimen. We defined a frozen section as being from the cancerous area if the biopsy site located within the tumor. Conversely, we defined a frozen section as being from the non-cancerous area if the biopsy site was outside the tumor (Fig. 1g).

The pathological diagnosis of a frozen section, i.e., “neoplasia,” “negative for neoplasia,” or “indefinite for neoplasia,” was compared to the final pathological diagnosis of the frozen section based on the mapping image of the formalin-fixed ESD specimen. We determined the frozen section diagnosis of “neoplasia” to be the final pathological diagnosis of “cancerous area,” or the frozen section diagnosis of “negative for neoplasia” to be the final pathological diagnosis of “non-cancerous area” as a correct diagnosis. Conversely, we determined the frozen section diagnosis of “neoplasia” to be the final pathological diagnosis of “non-cancerous area,” or the frozen section diagnosis of “negative for neoplasia” to be the final pathological diagnosis of “cancerous area” as a misdiagnosis. The following frozen sections were excluded from the analysis: (i) at least one pathologist judged the frozen section as inappropriate material for which histological diagnosis cannot be made, or (ii) the biopsy samples were obtained from the boundary between cancerous and non-cancerous area. The objectives of this study were to examine the accuracy of pathological diagnosis using frozen sections obtained via forceps biopsy and the consistency in diagnosis between the pathologists. In addition, we investigated the reasons for misdiagnosis or the diagnosis of “indefinite for neoplasia” by examining the formalin-fixed biopsy specimens.

### ESD procedure

ESD was performed with a conventional endoscope (GIF-H290Z, Olympus, Tokyo, Japan). We used magnifying endoscopy with narrow band imaging (NBI) together with white light endoscopy to identify the demarcation line of lesions. After recognizing the demarcation line, marking dots were placed around the lesion by coagulation using a needle knife. Submucosal injections were performed to lift the mucosal layer using glycerol (10% glycerol and 5% fructose; Chugai Pharmaceutical Co., Tokyo, Japan) or MucoUp (0.4% sodium hyaluronate; Johnson & Johnson, New Brunswick, New Jersey, USA) with a small amount of indigo carmine as injection solutions. Circumferential mucosal incisions and submucosal dissections were performed using an IT Knife 2 and an electrosurgical generator (VIO 300D; Erbe, Tubingen, Germany). The electrosurgical unit was set at a cutting current for mucosal incisions on Drycut mode, effect 4, 40W, and at a coagulating current for submucosal dissections on Soft Coagulation mode, effect 3, 30W.

### Preparation of frozen sections

Immediately after ESD, the fresh specimen was stretched and fixed on polystyrene with the mucosal surface facing upward, using mounting pins. Samples were collected from ESD fresh specimen using endoscopic biopsy forceps and used to prepare frozen sections. Biopsy samples were wrapped in saline-soaked gauze and promptly taken to the pathology laboratory. Samples were directly placed onto a metal chuck provided with Tissue-Tek O.C.T. Compound (Sakura Finetek, USA) and frozen using a freezing spray, PATH FREEZER (Matsunami Glass Ind, Osaka, Japan) in a cryostat set at −20 °C. The frozen tissue block was then mounted onto the microtome of the cryostat, thinly sliced to 4 μm, and placed on a glass slide. After the tissue sections were fixed with acetone at room temperature for 1 min, the slides were stained by immersing them in Gill’s Hematoxylin and Eosin for 1 min each. The slides were then dehydrated in alcohol, cleared in xylene, mounted with a coverslip and mounting medium, and used for pathological diagnosis. It took approximately 10 min, from biopsy sampling to completion, for the preparation of the frozen sections. Following this, the remaining samples were thawed, formalin-fixed, embedded in paraffin and stored. The formalin-fixed biopsy specimens were stained with hematoxylin and eosin and used for additional analysis to investigate the reasons for misdiagnosis or a diagnosis of “indefinite for neoplasia.”

### Statistical analysis

Diagnoses of frozen sections were compared to the corresponding pathological diagnoses of ESD specimens to evaluate the accuracy of frozen section assessments, as described previously. Inter-rater concordance was analyzed using the percentage of raw agreement and Cohen’s kappa coefficient [30]. The degree of diagnostic concordance between pathologists was evaluated by the Landis and Koch criteria [31]. The Fisher exact test was used to compare categorical variables and statistical significance was set at *p* < 0.05. All statistical analyses were performed using the STATA 17.0 software program (Stata Corporation, College Station, TX, USA) or the JMP Pro 15.1.0 software program (SAS Institute, Cary, NC, USA).

## Results

### Clinicopathological features

The 32 patients with early gastric cancer lesions included 25 men and seven women. The median patient age was 76 years (range 41 – 94 years). With regard to *H. pylori* infection status; three patients (9.4%) were uninfected, four (12.5%) were currently infected, 12 (37.5%) were post-eradication, and 13 (40.6%) had *H. pylori* which spontaneously disappeared. The median size of the tumors treated with ESD was 11 mm (range: 4 – 38 mm), and the median size of the ESD specimens was 34 mm (range: 17– 58 mm). The distribution of the lesion locations was as follows: five (15.6%) in the upper third, 12 (37.5%) in the middle third, and 15 (46.9%) in the lower third. Macroscopically, 12 lesions (37.5%) were elevated, five (15.6%) were flat, and 15 (46.9%) were depressed. En bloc resection was achieved for all 32 lesions (100%) (Table 1).

**Table 1:** Patient and tumor characteristics

Variable	Values, *n* = 32	
Sex		
Male	25	(78.1%)
Female	7	(21.9%)
Median age, year (range)	76	(41–94)
*H. pylori* infection status		
Non-infection	3	(9.4%)
Current infection	4	(12.5%)
Post-eradication	12	(37.5%)
Spontaneous-disappearance	13	(40.6%)
Tumor location		
Upper third	5	(15.6%)
Middle third	12	(37.5%)
Lower third	15	(46.9%)
Median tumor size, mm (range)	11	(4–38)
Median ESD specimen size, mm (range)	34	(17–58)
Microscopic type		
Elevated (0-I or 0-IIa)	12	(37.5%)
Flat (0-IIb)	5	(15.6%)
Depressed (0-IIc)	15	(46.9%)
Histologic type		
Pap	0	(0.0%)
Tub1	24	(75.0%)
Tub2	5	(15.6%)
Por	1	(3.1%)
Sig	2	(6.3%)
Invasion depth		
M	29	(90.6%)
SM	2	(6.3%)
MP	1	(3.1%)
Ulcerative finding		
Present	3	(9.4%)
Absent	29	(90.6%)
Lymphovascular infiltration		
Present	2	(6.3%)
Absent	30	(93.8%)
Lateral margin		
Positive	0	(0.0%)
Negative	32	(100%)
Vertical margin		
Positive	2	(6.3%)
Negative	30	(93.8%)
En bloc resection	32	(100.0%)

*H. pylori, Helicobacter pylori*; ESD, endoscopic submucosal dissection; Pap, papillary adenocarcinoma; Tub1, well-differentiated tubular adenocarcinoma; Tub2, moderately differentiated tubular adenocarcinoma; Por, poorly differentiated adenocarcinoma; Sig, signet ring cell carcinoma; M, mucosa; SM, submucosa; MP, muscularis propria

### Histological analysis of ESD specimens

All 32 lesions were resected with negative lateral margins and two lesions (6.3%) with positive vertical margins. Twenty-nine lesions (90.6%) were differentiated-type adenocarcinomas: 24 (75.0%) tub1 and five (15.6%) tub2. Three lesions (9.4%) were undifferentiated-type adenocarcinomas: one (3.1%) por and two (6.3%) sig. (Table 1).

### Pathological images of frozen sections

**Fig. 2:**
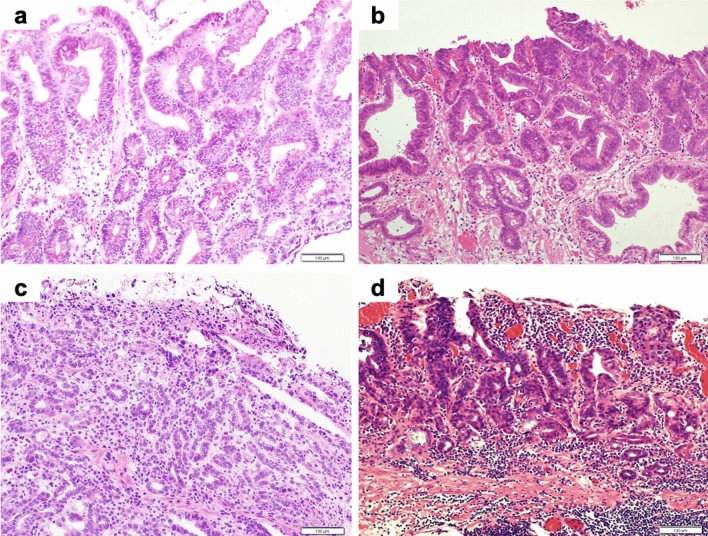
Representative pathological images of frozen sections and ESD specimens of differentiated-type carcinoma (hematoxylin and eosin staining). **a, b** Well-differentiated tubular adenocarcinoma (tub1), **a** Frozen section (× 100); **b** ESD specimen (× 100). **c, d** Moderately differentiated tubular adenocarcinoma (tub2); **c** Frozen section (× 100); **d** ESD specimen (× 100)

**Fig. 3:**
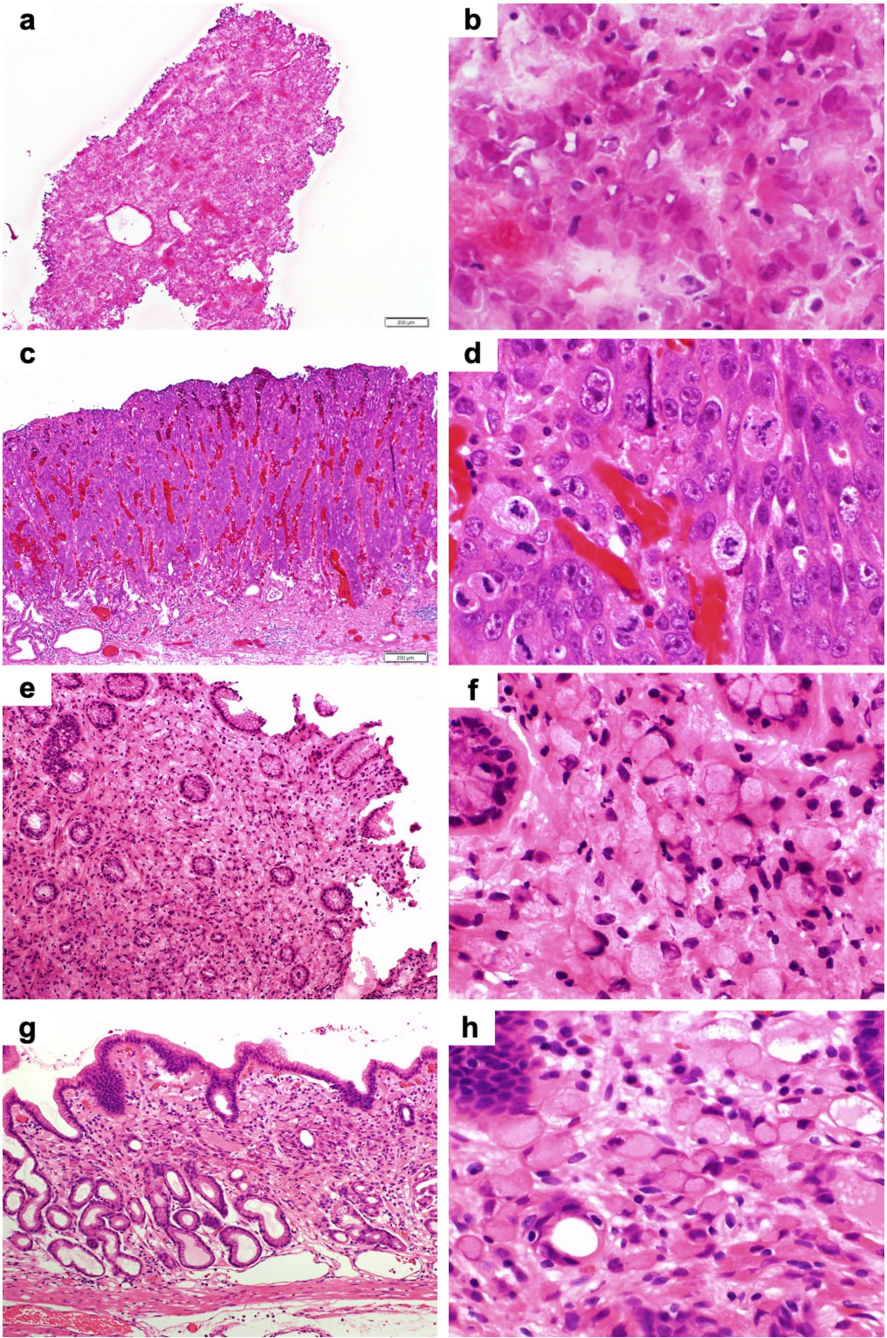
Representative pathological images of frozen sections and ESD specimens of undifferentiated-type carcinoma (hematoxylin and eosin staining). **a–d** Poorly differentiated adenocarcinoma (por2 > tub2); **a, b** Frozen section (**a** × 40, **b** × 400); **c, d** ESD specimen (**c** × 40, **d** × 400). **e−h** Signet ring cell carcinoma (sig); **e, f** Frozen section (**e** × 100, **f** × 400); **g, h** ESD specimen (**g** × 100, **h** × 400)

**Fig. 4:**
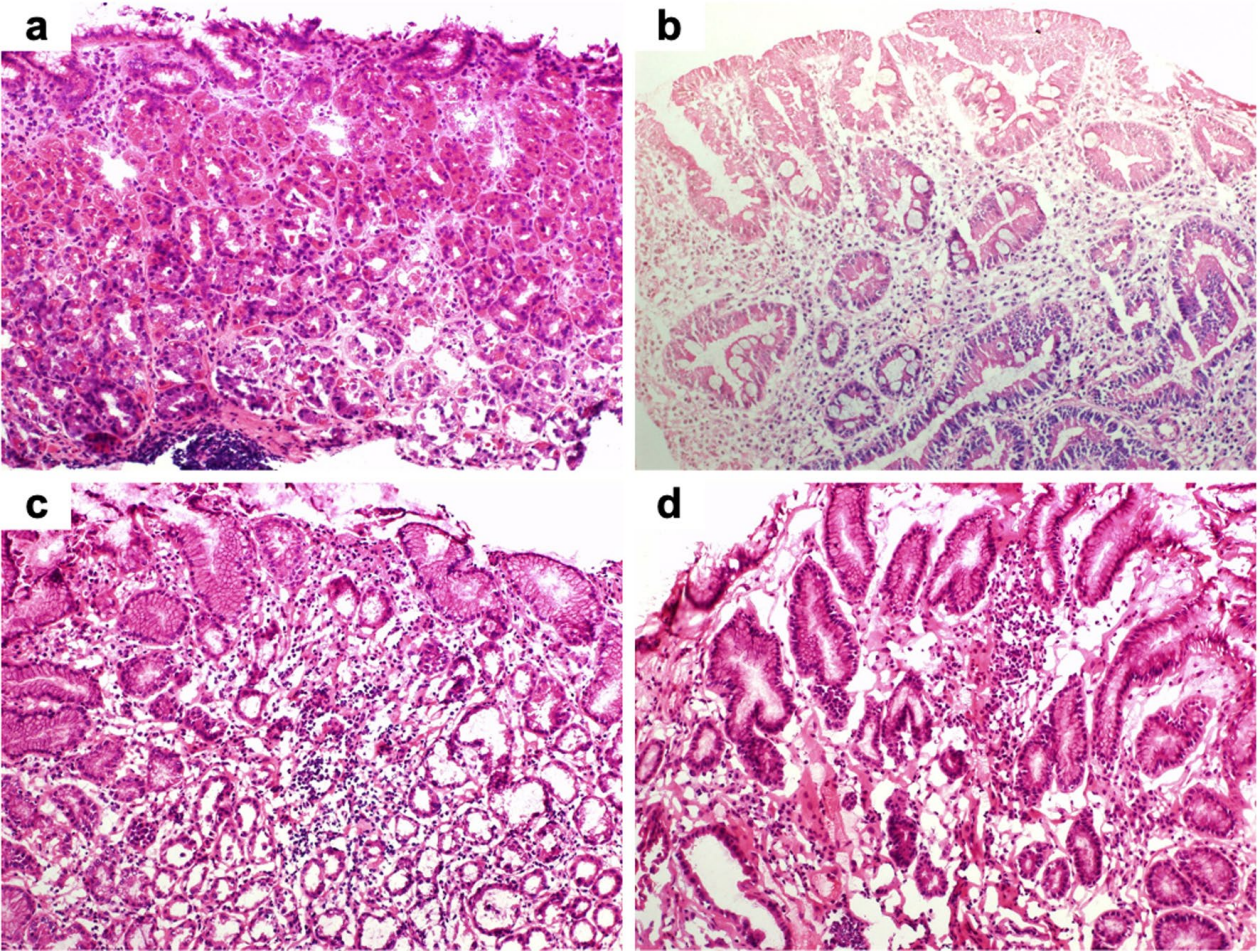
Representative pathological images of frozen sections of normal gastric mucosa (hematoxylin and eosin staining). **a** Fundic gland mucosa without atrophy and intestinal neoplasia (× 100). **b** Fundic gland mucosa with severe atrophy (× 100). **c** Fundic gland mucosa with severe atrophy and severe intestinal neoplasia (× 100). **d** Pyloric gland mucosa with mild atrophy (× 100)

Representative pathology of the frozen sections from cancerous areas compared to the ESD specimens is shown in Figs. 2, 3. Figure 4 shows the frozen sections from non-cancerous areas. Although frozen sections showed freezing artifacts and a decreased intensity of the stain compared with formalin-fixed ESD specimens, correct diagnoses can be made.

### Accuracy of pathological diagnosis of frozen sections

Two pathologists independently evaluated 149 frozen sections. Eleven frozen sections were judged to be inappropriate by at least one pathologist: nine frozen sections had severe artifacts and two frozen sections lacked epithelial components. Of the 149 frozen sections prepared, 10 were determined to have been obtained from the boundaries between the cancerous and the non-cancerous area. Two frozen sections met both of the exclusion criteria (Table S1). Hence, 19 frozen sections were excluded from the analysis, and the diagnostic accuracy of 130 frozen sections was examined by two different pathologists. Of the 130 frozen sections, 35 were obtained from cancerous areas and 95 were obtained from non-cancerous areas. The histologic type of the lesions and the number of biopsy samples per patient are shown in Table S2. As shown in Table 2, the diagnostic accuracies of the frozen sections by pathologists A and B were 98.5% (128/130) and 94.6% (123/130), respectively. The frozen sections from cancerous areas were correctly diagnosed as “neoplasia” by the two pathologists in 97.1% (34/35) and 82.9% (29/35) of the sections, respectively. Those from non-cancerous areas were correctly diagnosed as “negative for neoplasia” in 98.9% (94/95) and 98.9% (94/95) of the sections, respectively. The frozen sections from cancerous areas were diagnosed as “indefinite for neoplasia” in 0% (0/35) and 17.1% (6/35) of the sections, and misdiagnosed as “negative for neoplasia” in 2.9% (1/35) and 0% (0/35) of the sections, respectively. On the other hand, the frozen sections from non-cancerous areas were diagnosed as “indefinite for neoplasia” in 1.1% (1/95) and 1.1% (1/95) of the sections, respectively, and no frozen sections from non-cancerous areas were misdiagnosed as “neoplasia” by either pathologist. The cross-tabulation table of pathological diagnoses of the frozen sections by pathologists is shown in Table 3. The percentage of raw agreement between the pathologists was 93.8% and the Cohen’s kappa coefficient was 0.851 (95% CI: 0.837–0.864), indicating an “almost perfect” agreement between the two according to the Landis and Koch criteria. There were no significant differences in the diagnostic accuracy of frozen sections between the two pathologists for *H. pylori* infection status (post eradication vs. non-post eradication), lesion location (upper third vs. middle or lower third), lesion morphology (flat vs. non-flat type), or histologic type (undifferentiated vs. differentiated type).

**Table 2:** Pathological diagnosis of the frozen section by pathologists A and B

		The final pathology compared to ESD specimen	
		Cancerous area, *n* = 35	Non-cancerous area, *n* = 95
Pathologist A	Neoplasia	34 (97.1%)	0 (0%)
	Indefinite	0 (0%)	1 (1.1%)
	Negative for neoplasia	1 (2.9%)	94 (98.9%)
Pathologist B	Neoplasia	29 (82.9%)	0 (0%)
	Indefinite	6 (17.1%)	1 (1.1%)
	Negative for neoplasia	0 (0%)	94 (98.9%)

**Table 3:** Cross-tabulation table of pathological diagnosis of frozen section

		Pathologist B			
		Neoplasia	Indefinite	Negative for neoplasia	Total, *n*
Pathologist A	Neoplasia	29	5	0	34
	Indefinite	0	0	1	1
	Negative for neoplasia	0	2	93	95
	Total, *n*	29	7	94	130

### Frozen sections that were misdiagnosed or diagnosed as indefinite for neoplasia

Of the 130 frozen sections, six from cancerous areas and two from non-cancerous areas were misdiagnosed or diagnosed as “indefinite for neoplasia” by at least one pathologist. We made formalin-fixed specimens from these biopsy samples and both pathologists evaluated the specimens to reveal the reasons for the incorrect diagnosis by comparing the pathology findings of the frozen sections, the fixed biopsy specimens, and the ESD specimens (Table 4, Cases #1–8).

**Table 4:** Frozen sections misdiagnosed or diagnosed as “indefinite for neoplasia”

Case	Final pathology compared to ESD specimen	Pathologist	Frozen section		Formalin-fixed biopsy specimen		Tumor characteristics			
			Diagnosis	Reasons for incorrect diagnosis	Diagnosis	Reasons for incorrect diagnosis	Histologic type	Microscopic type	Location	*H. pylori* infection status
#1	Cancerous area	A	Negative for neoplasia	Freezing artifacts, inflammation, and tumor cells with mild nuclear atypia	Indefinite	Inflammation, and tumor cells with mild nuclear atypia	tub1	Elevated	L	Spontaneous disappearance
		B	Indefinite		Indefinite					
#2	Cancerous area	A	Neoplasia	Freezing artifacts, and small volume of tissue sample	Neoplasia		tub1	Depressed	L	Post eradication
		B	Indefinite		Neoplasia					
#3	Cancerous area	A	Neoplasia	Freezing artifacts, and tumor cells with mild nuclear atypia and minimal architectural distortion	Neoplasia		tub1 > tub2	Elevated	L	Post eradication
		B	Indefinite		Neoplasia					
#4	Cancerous area	A	Neoplasia	Freezing artifacts, small volume of tissue sample, and tumor cells with mild nuclear atypia	Indefinite	Artifacts, and small volume of tissue sample	tub1 > tub2	Elevated	M	Spontaneous disappearance
		B	Indefinite		Neoplasia					
#5	Cancerous area	A	Neoplasia	Freezing artifacts, and insufficient staining	Negative for neoplasia	Artifacts	tub1 > tub2	Elevated	M	Spontaneous disappearance
		B	Indefinite		Indefinite					
#6	Cancerous area	A	Neoplasia	Inflammation	Indefinite	Artifacts, and small volume of the tissue sample	tub1 > pap	Elevated	M	Spontaneous disappearance
		B	Indefinite		Indefinite					
#7	Non-cancerous area	A	Negative for neoplasia	Freezing artifacts, and improper horizontal cutting	Negative for neoplasia		tub2 > tub1	Elevated	U	Spontaneous disappearance
		B	Indefinite		Negative for neoplasia					
#8	Non-cancerous area	A	Indefinite	Thermal damage	Negative for neoplasia	Thermal damage	tub2 > tub1	Depressed	M	Current infection
		B	Negative for neoplasia		Indefinite					

ESD, endoscopic submucosal dissection; tub1, well-differentiated tubular adenocarcinoma; tub2, moderately differentiated tubular adenocarcinoma; pap, papillary adenocarcinoma; L, lower third; M, middle third; U, upper third; *H. pylori, Helicobacter pylori*

In one of the six frozen sections from the cancerous areas, pathologist A misdiagnosed it as “negative for neoplasia” and pathologist B diagnosed it as “indefinite for neoplasia” (Table 4, Case #1). Both pathologists diagnosed the fixed biopsy specimen as “indefinite for neoplasia.” Fig. S1 shows the pathological images of the frozen section, the fixed biopsy specimen, and the ESD specimen of Case #1. There were inflammatory cell infiltrates, freezing artifacts, and a well-differentiated tubular adenocarcinoma with mild nuclear atypia, which were the reasons for the misdiagnosis of the frozen section. In the remaining five frozen sections from cancerous areas, pathologist A correctly diagnosed them as “neoplasia,” whereas, pathologist B diagnosed them as “indefinite for neoplasia” (Table 4, Cases #2–6). The incorrect diagnosis resulted from freezing artifacts, insufficient staining results, small volume of tissue samples, inflammation, and/or a well-differentiated adenocarcinoma with mild nuclear atypia (Figs. S2, S3). Among the frozen sections from cancerous areas that were incorrectly diagnosed, both pathologists made a correct diagnosis of “neoplasia” in only two fixed biopsy specimens. On the other hand, no frozen sections from non-cancerous areas were misdiagnosed as “neoplasia.” Additionally, two frozen sections were diagnosed as “indefinite for neoplasia” by at least one pathologist (Table 4, Cases #7–8). Improper cutting (horizontal to the mucosa) and thermal damage to the tissues during ESD made it difficult for the pathologist to make the correct diagnosis of “negative for neoplasia” (Figs. S4, S5). Among the two fixed biopsy specimens from non-cancerous areas, only one specimen was correctly diagnosed as “negative for neoplasia” by both the pathologists.

## Discussion

The purpose of this study was to reveal the diagnostic accuracy of frozen section biopsy for early gastric cancer to assess margins during gastric ESD. The diagnostic accuracies of frozen section biopsies by two different pathologists were 98.5 and 94.6%, respectively, with high inter-pathologist concordance. Frozen sections from the cancerous areas were correctly diagnosed by the two pathologists in 97.1 and 82.9% of the sections, and misdiagnosed in 2.9 and 0% of the sections, respectively. Frozen sections from the non-cancerous areas were correctly diagnosed in 98.9 and 98.9% of the sections, and misdiagnosed in 0 and 0% of the sections, respectively. The diagnostic accuracy of frozen sections by surgical IOC has been reported to be 95.8–99.4%, with misdiagnosis of cancerous frozen sections reportedly occurring in 0.2–26.1% [26, 32–35]. In the present study, the results were comparable to those previously reported, despite the pathological diagnoses being made with a small amount of tissue obtained using endoscopic biopsy forceps. Therefore, we suggest that the pathological diagnosis of frozen section biopsy is reliable and can be applied to determine the extent of lesions during ESD.

The diagnosis of “indefinite for neoplasia” by pathologists A and B, accounting for 0.8% (1/130) and 5.4% (7/130) of the sections, respectively, resulted from two factors, i.e., quality of the frozen sections and nature of the lesions. The reasons for inappropriate quality of frozen sections included freezing artifacts, insufficient staining, small volume of tissue, and denaturation of tissue due to ESD. The nature of the lesion that hampered pathologists’ diagnoses included inflammation-induced atypia similar to neoplastic cells, inflammatory cell infiltration to the stroma, and the inconspicuous nuclear atypia of well-differentiated tubular adenocarcinomas.

With inflammatory cell infiltration into the stroma, cancer with mild nuclear atypia and minimal architectural distortion can be confused with reactive atypia induced by inflammation. Injection of fluid into the submucosal layer, thermocoagulation, contact with endoscopic attachment, and decreased blood flow to the tissue were considered to result in denaturation of the tissue. In addition, formation of ice crystals as water freezes in the tissue causing cytoclasis and vacuolation, leads to unavoidable freezing artifacts and insufficient staining during frozen section preparation.

In this study, 75% of the frozen sections diagnosed as “indefinite for neoplasia” were biopsied from cancerous areas, and in about half of them, histopathological characteristics of a well-differentiated tubular adenocarcinoma with mild nuclear atypia made the correct diagnosis difficult. These results suggest that, when frozen sections were diagnosed as “indefinite for neoplasia” during ESD, the biopsy samples were more likely to contain cancer cells. In cases where conventional endoscopic biopsy specimens fixed with formalin are pathologically diagnosed as “indefinite for neoplasia,” assessment with re-biopsy specimens is generally recommended [36, 37]. Consequently, if the rapid pathological diagnosis using frozen section biopsy during ESD is “indefinite for neoplasia,” reexamination with another sample can be done.

No frozen sections from non-cancerous areas were misdiagnosed as “neoplasia.” Meanwhile, only one section from a cancerous area was misdiagnosed as “negative for neoplasia.” This misdiagnosis resulted from the artifacts introduced during frozen section preparation and the lesion characteristics of inflammation and those of a well-differentiated tubular adenocarcinoma with mild nuclear atypia. However, the endoscopic image showed an elevated type (0-I) lesion with a well-defined margin, with the lateral extent of the lesion easily recognized optically. In addition, the other pathologist diagnosed the frozen section as “indefinite for neoplasia,” suggesting that misdiagnosis of frozen sections may be avoided by discussion between two or more pathologists. In rapid frozen section diagnosis of a well-differentiated tubular adenocarcinoma with mild nuclear atypia and minimal architectural distortion, pathological information (histologic subtype, degree of nuclear atypia, and structural distortion) based on fixed biopsy specimens of the lesion taken during preoperative esophagogastroduodenoscopy may help the pathologist make an accurate diagnosis.

Furthermore, in this study, frozen section biopsy diagnosis was reliable regardless of the predictive factors for inaccurate determination of the lateral extent of early gastric cancer, i.e., cancer developed after eradication therapy of *Helicobacter pylori* (*H. pylori*), location in the upper third of the stomach, flat type cancer, and undifferentiated-type cancer.

In surgical IOC, diffuse-type or signet ring cell carcinomas have been reported as risk factors for the misdiagnosis of frozen sections [33, 34, 38]. A single institutional study [34] examined 144 frozen sections of 81 patients with esophagogastric adenocarcinoma who underwent operative intervention and demonstrated that the diagnostic accuracy of frozen sections was 95.8% (138/144) and the frequency of misdiagnoses of cancerous frozen sections was 26.1% (6/23). These misdiagnoses were due to missed signet ring cells or freezing artifacts precluding an accurate diagnosis. On hematoxylin and eosin staining, signet ring cells that produce mucin can be confused with macrophages containing mucoprotein, and conversely, some cells that have less mucin may be difficult to distinguish from lymphocytes. In IOC using frozen sections, providing the pathologist with the appropriate clinical information assists in making a correct pathological diagnosis [39]. In the present study, the pathologists were given information on differentiated- or undifferentiated-type cancer based on the pathology results of the preoperative biopsy. Some studies have proposed that rapid special immunohistochemical staining such as with cytokeratin and mucin, in combination with frozen sections, aided difficult cases in surgical IOC [40, 41]. Thus, such staining can be an option for rapid frozen section diagnosis during ESD.

In clinical practice, the goal of frozen section biopsy during ESD is to determine the lateral extent of the lesion for accurate excision. Therefore, it is important that biopsies from non-cancerous areas be accurately diagnosed as “negative for neoplasia” in frozen section analyses. The results of the present study showed that no frozen section from a non-cancerous area was misdiagnosed as “neoplasia”, regardless of the pathologist’s experience, which revealed that pathological diagnosis using frozen section biopsy can be applied in determining the lateral extent of cancer during ESD. Although it takes approximately 15 min from preparation to diagnosis of frozen sections, the procedure does not require specialized equipment and can be performed repeatedly.

Frozen section biopsy during ESD may help avoid unnecessary, extensive dissection, which may consequently reduce complications such as bleeding and perforation associated with ESD.

This study has several limitations. First, this was a single-institutional study with a small number of cases, with particularly limited patients with undifferentiated-type carcinomas. Second, from an ethical point of view, especially for patient safety, the biopsy samples for frozen sections in our study were not obtained during ESD but after the completion of ESD. Fresh specimens resected by ESD might have been affected by ESD procedure-related tissue damage. Third, since we adopted a method for preparing frozen sections with surgically resected tissue, the method for frozen section biopsy in this study might not be optimized.

In conclusion, to the best of our knowledge, this is the first study to examine the diagnostic accuracy of frozen section biopsy for early gastric cancer. It has revealed that pathological diagnosis of frozen section biopsy had high diagnostic accuracy and good diagnostic concordance between pathologists. We believe that the results of this study can be applied to assess the lateral margins of lesions during ESD and contribute to improving treatment outcomes.

## Supplementary Information

Below is the link to the electronic supplementary material.

Supplementary file1 (DOCX 15550 KB)
