# Supplementary material for: Diagnostic accuracy of frozen section biopsy for early gastric cancer extent during endoscopic submucosal dissection: a prospective study
Source: Surg Endosc. 2023 May 22;37(9):6736–48. doi: 10.1007/s00464-023-10100-2 (PMC10462503; doi:10.1007/s00464-023-10100-2)
Supplement: Supplementary file 1 — Supplementary file1 (DOCX 15550 KB) [file 464_2023_10100_MOESM1_ESM.docx]

**Supplementary Methods**

*Helicobacter Pylori (H. pylori) infection status*

Non-cancer areas of the endoscopic submucosal dissection specimens were examined for the presence and grade of mononuclear cell infiltrate (i.e., chronic inflammation), neutrophil infiltrate (i.e., activity of inflammatory), glandular atrophy, intestinal metaplasia, and *H. pylori* infection and scored according to the updated Sydney System. *H. pylori* infection status was determined based on the presence or absence of endoscopic mucosal atrophy, serum IgG antibody tests, and microscopic observation, and was classified as follows; (i) Non-infection: no history of *H. pylori* eradication, absence of endoscopic mucosal atrophy, a negative serum antibody test, and absence of *H. pylori* on microscopic observation, (ii) Current infection: presence of endoscopic mucosal atrophy, and a positive serum antibody test or presence of *H. pylori* on microscopic observation, (iii) Post-eradication: history of successful *H. pylori* eradication and absence of *H. pylori* on microscopic observation, and (iv) Spontaneous-disappearance: no history of *H. pylori* eradication, presence of endoscopic mucosal atrophy, a negative serum antibody test, and absence of *H. pylori* on microscopic observation.

**Supplementary Table 1.** Frozen sections excluded from analysis

| Case | Reason for exclusion:  (Inappropriate^†^ or Boundary^‡^) | Final pathology compared to ESD specimen | Pathological diagnosis  of frozen section | | Tumor characteristics | | | |
| --- | --- | --- | --- | --- | --- | --- | --- | --- |
|  |  |  | Pathologist A | Pathologist B | Histologic type | Microscopic type | Location | H. pylori infection status |
| 1 | Inappropriate | Cancer | Inappropriate | Neoplasia | tub2 | Depressed | M | Spontaneous disappearance |
| 2 | Inappropriate | Cancer | Inappropriate | Indefinite | tub1 | Depressed | M | Non-infection |
| 3 | Inappropriate | Cancer | Inappropriate | Inappropriate | tub1 | Depressed | M | Post eradication |
| 4 | Inappropriate | Non-cancer | Negative for neoplasia | Inappropriate | tub1 > tub2 | Depressed | L | Post eradication |
| 5 | Inappropriate | Non-cancer | Inappropriate | Negative for neoplasia | tub2 | Depressed | M | Spontaneous disappearance |
| 6 | Inappropriate | Non-cancer | Inappropriate | Inappropriate | tub1 > tub2 | Depressed | L | Post eradication |
| 7 | Inappropriate | Non-cancer | Inappropriate | Inappropriate | tub1 | Elevated | M | Spontaneous disappearance |
| 8 | Inappropriate | Non-cancer | Inappropriate | Inappropriate | tub1 | Depressed | U | Spontaneous disappearance |
| 9 | Inappropriate | Non-cancer | Inappropriate | Inappropriate | tub1 | Depressed | M | Post eradication |
| 10 | Inappropriate, Boundary | Indeterminable | Indefinite | Inappropriate | sig > por | Elevated | M | Post eradication |
| 11 | Inappropriate, Boundary | Indeterminable | Negative for neoplasia | Inappropriate | por2 > tub2 | Elevated | U | Post eradication |
| 12 | Boundary | Indeterminable | Indefinite | Indefinite | tub2 > sig | Depressed | L | Non-infection |
| 13 | Boundary | Indeterminable | Indefinite | Neoplasia | tub2 > sig | Depressed | L | Non-infection |
| 14 | Boundary | Indeterminable | Neoplasia | Indefinite | tub1 | Flat | M | Current infection |
| 15 | Boundary | Indeterminable | Indefinite | Indefinite | tub1 > tub2 | Flat | L | Current infection |
| 16 | Boundary | Indeterminable | Indefinite | Indefinite | sig | Flat | U | Non-infection |
| 17 | Boundary | Indeterminable | Negative for neoplasia | Indefinite | tub1 > pap,tub2 | Elevated | L | Post eradication |
| 18 | Boundary | Indeterminable | Negative for neoplasia | Indefinite | tub1 | Depressed | U | Spontaneous disappearance |
| 19 | Boundary | Indeterminable | Negative for neoplasia | Negative for neoplasia | tub1 > tub2 | Depressed | L | Post eradication |
| † At least one pathologist judged the frozen section as an inappropriate material for which histological diagnosis cannot be made. | | | | | | | | |
| ‡ The biopsy sample was obtained from the boundary between the cancerous and non-cancerous areas. | | | | | | | | |
| ESD, endoscopic submucosal dissection; tub1, well-differentiated tubular adenocarcinoma; tub2, moderately-differentiated tubular adenocarcinoma; por2, poorly-differentiated adenocarcinoma (non-solid type); sig, signet ring cell carcinoma; L, lower third; M, middle third; U, upper third; *H. pylori,* *Helicobacter Pylori.* | | | | | | | | |

**Supplementary Table 2**. Histologic type of the lesion and number of biopsy samples per patient

| Number of biopsy samples  per ESD specimen | | Number of ESD specimens | Histologic type according to the final pathological diagnosis of the ESD specimen | | | | |
| --- | --- | --- | --- | --- | --- | --- | --- |
| from cancer area | from non-cancer area |  | pap | tub1 | tub2 | por | sig |
| 0 | 2 | 2 | 0 | 2 | 0 | 0 | 0 |
| 0 | 3 | 3 | 0 | 2 | 1 | 0 | 0 |
| 0 | 4 | 3 | 0 | 3 | 0 | 0 | 0 |
| 0 | 5 | 3 | 0 | 2 | 0 | 0 | 1 |
| 1 | 0 | 1 | 0 | 0 | 0 | 1 | 0 |
| 1 | 1 | 1 | 0 | 1 | 0 | 0 | 0 |
| 1 | 3 | 3 | 0 | 2 | 0 | 0 | 1 |
| 1 | 4 | 4 | 0 | 2 | 2 | 0 | 0 |
| 2 | 1 | 1 | 0 | 0 | 1 | 0 | 0 |
| 2 | 2 | 4 | 0 | 4 | 0 | 0 | 0 |
| 2 | 3 | 4 | 0 | 3 | 1 | 0 | 0 |
| 2 | 4 | 1 | 0 | 1 | 0 | 0 | 0 |
| 3 | 2 | 2 | 0 | 2 | 0 | 0 | 0 |
| Total | | 32 | 0 | 24 | 5 | 1 | 2 |
| ESD, endoscopic submucosal dissection; pap, papillary adenocarcinoma; tub1, well-differentiated tubular adenocarcinoma; tub2, moderately-differentiated tubular adenocarcinoma; por, poorly-differentiated adenocarcinoma; sig, signet ring cell carcinoma; | | | | | | | |

**Supplementary Figure 1.**

**
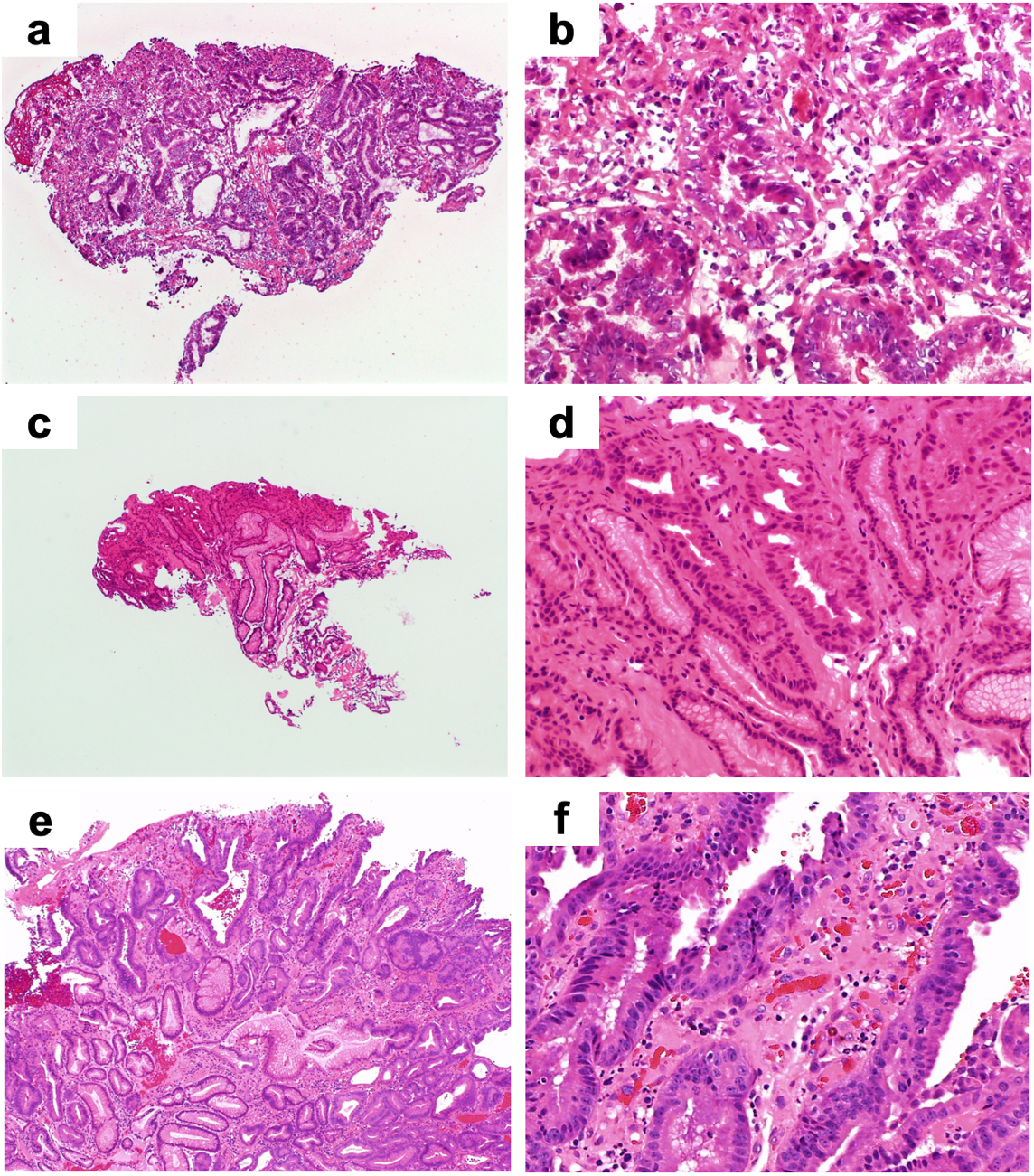
**

Pathological images of the cancerous frozen section misdiagnosed as “negative for neoplasia” (Case #1 in Table 4).

**a-b** The frozen section shows atypical cells, abundant inflammatory cells infiltrating to the stroma, and freezing artifacts, which made the atypical cells difficult to differentiate between cancer cells and reactive epithelium induced by inflammation (HE, **a** x40; **b** x200). **c-d** On the fixed biopsy specimen, freezing artifacts are absent, while mild nuclear atypical cells, and inflammatory cell infiltration of stroma, make it difficult to determine whether cancer cells are observed (HE, **c** x40; **d** x200). **e-f** The ESD specimen shows well-differentiated tubular adenocarcinoma (tub1) with mild nuclear atypia and abundant inflammation (HE, **e** x40; **f** x200).

**Supplementary Figure 2.**


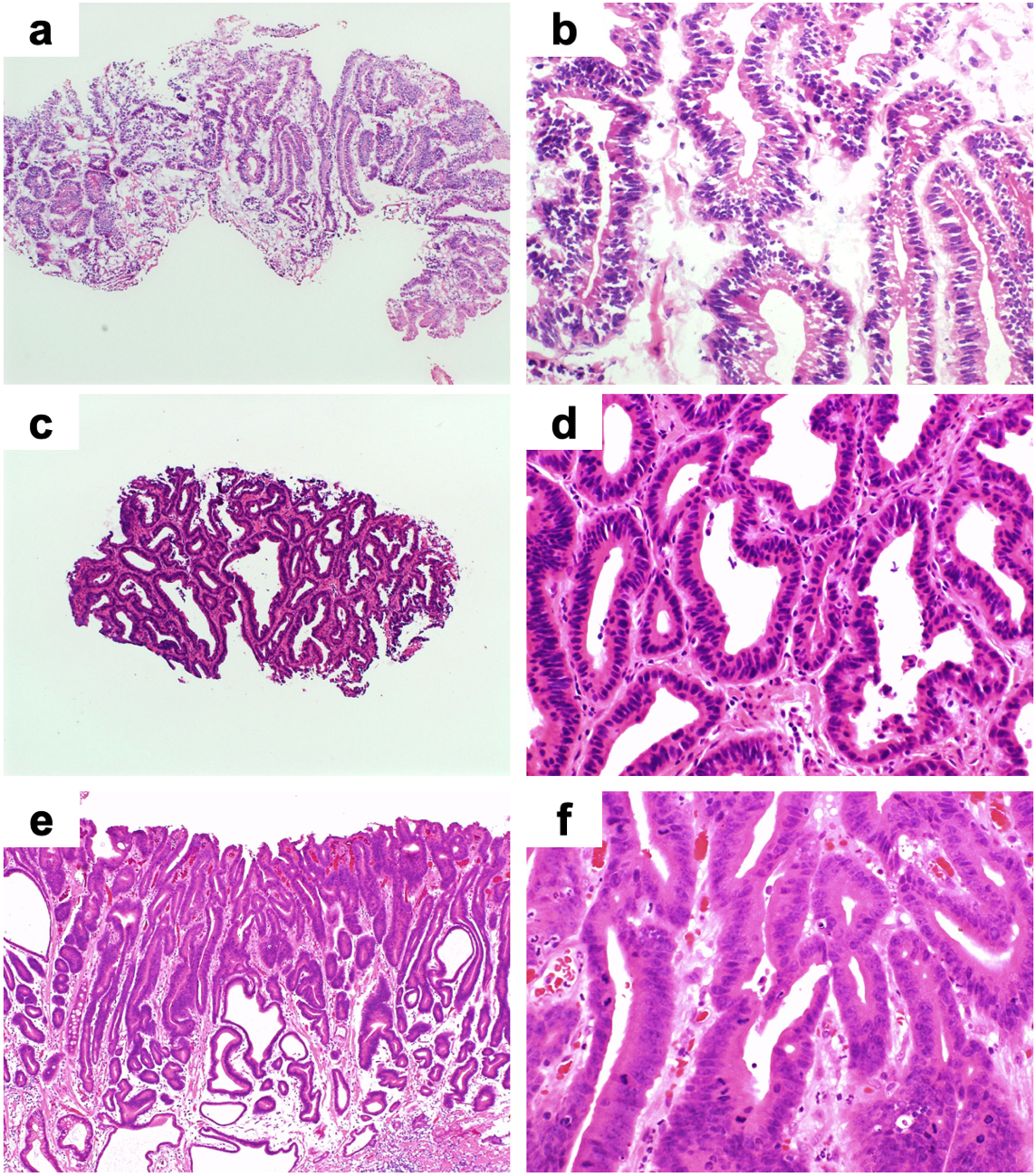


Pathological images of the cancerous frozen section misdiagnosed as “indefinite for neoplasia” (Case #3 in Table 4).

**a-b** The frozen section shows mild nuclear atypical cells, minimal architectural distortion and freezing artifacts, making it difficult to differentiate cancer cells from intestinal metaplasia (HE, **a** x40; **b** x200). **c-d** The fixed biopsy specimen provides adequately stained images without freezing artifacts, and shows dense tumor ducts consisting of epithelial cells with mild nuclear atypia (HE, **c** x40; **d** x200). **e-f** The ESD specimen shows well-differentiated tubular adenocarcinoma (tub1) with mild nuclear atypia and minimal architectural distortion (HE, **e** x40; **f** x200).

**Supplementary Figure 3.**


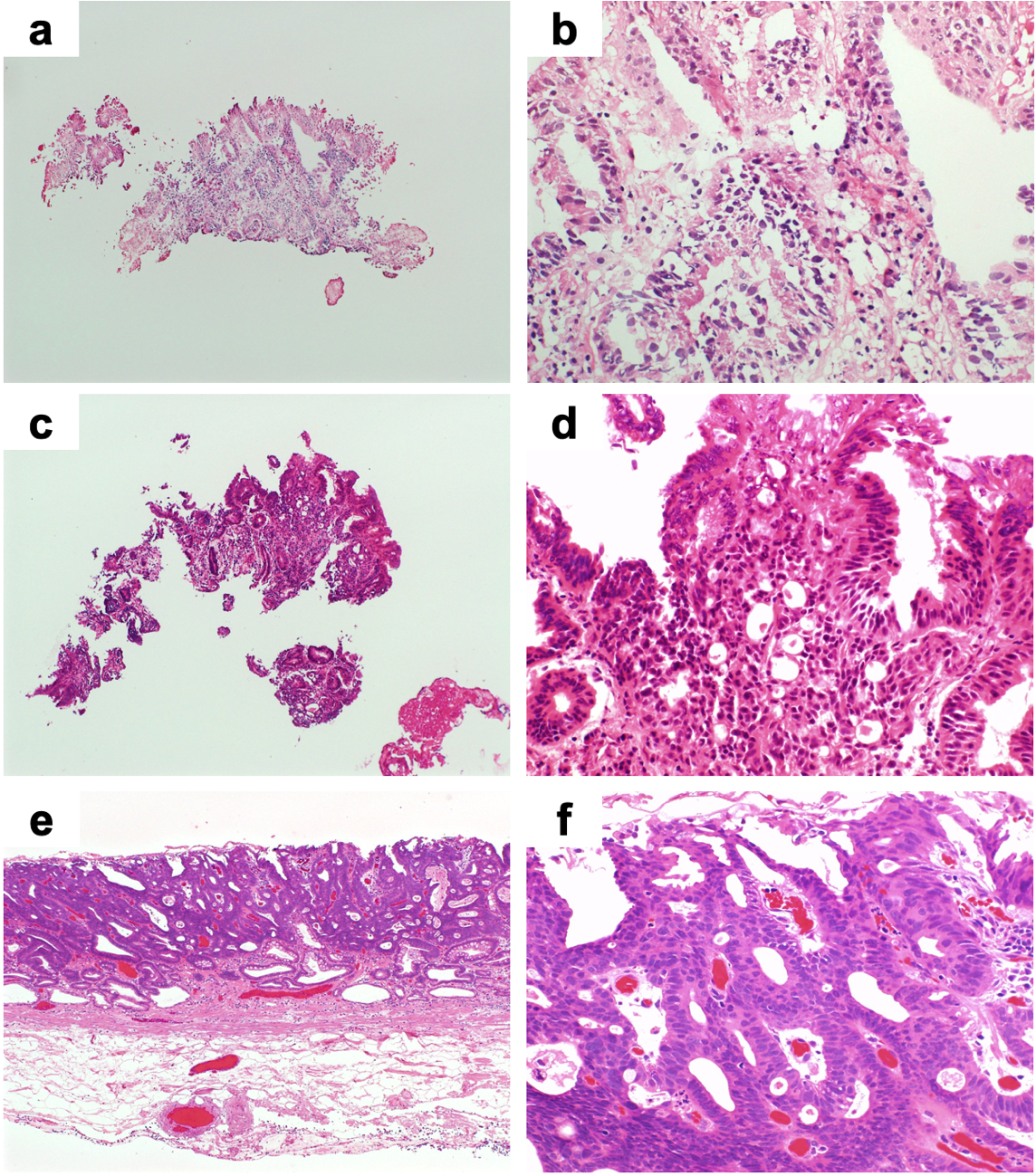


Pathological images of the cancerous frozen section misdiagnosed as “indefinite for neoplasia” (Case #4 in Table 4).

**a-b** The frozen section shows a small volume of tissue, freezing artifacts, insufficient staining, and epithelial cells without atypia in the topmost surface layer of the mucosa, with unclear gland duct architecture and mild nuclear atypia in the deep layer of the mucosa (HE, **a** x40; **b** x200). **c-d** The fixed biopsy specimen has less freezing artifacts and an increased intensity of stain that helps clarify cancer cells with mild nuclear atypia and the formation of irregular branching small ducts and cribriform ducts in the deep layer of the mucosa (HE, **c** x40; **d** x200). **e-f** The ESD specimen shows well- and moderately-differentiated tubular adenocarcinoma with similar morphology to the fixed biopsy specimen (tub1 > tub2) (HE, **e** x40; **f** x200).

**Supplementary Figure 4.**


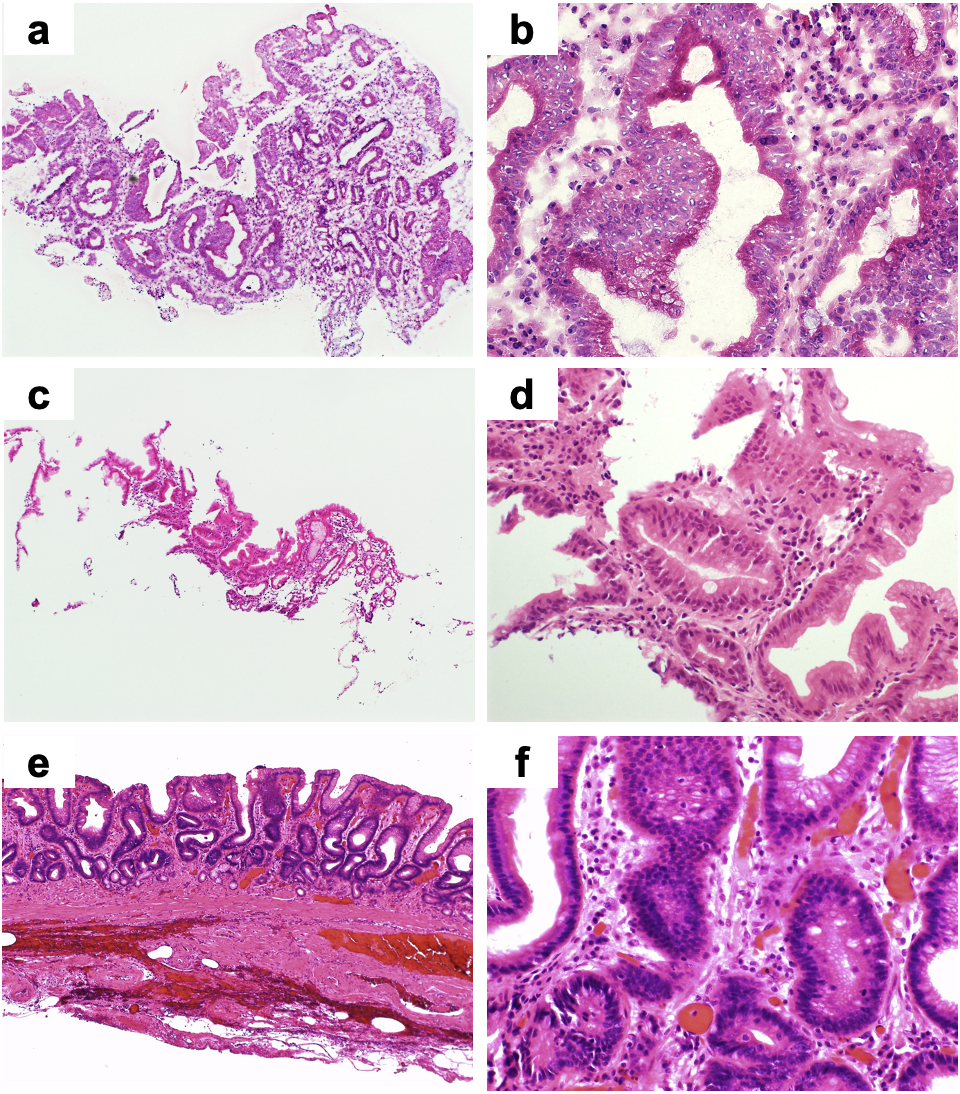


Pathological images of the non-cancerous frozen section diagnosed as “indefinite for neoplasia” (Case #7 in Table 4).

**a-b** The frozen section shows freezing artifacts and a horizontal cutting finding to the mucosa, especially the proliferative cell zone of intestinal metaplasia in the left half of the image (HE, **a** x40; **b** x200). **c-d** The fixed biopsy specimen shows the surface layer of metaplastic intestinal mucosa (HE, **c** x40; **d** x200). **e-f** The ESD specimen shows fundic gland mucosa with moderate atrophy and mild intestinal metaplasia. Incorrect diagnosis in the frozen section resulted from that the atypical and stratified nuclei in the proliferative cell zone mimicking those seen in cancer cells making it difficult to determine proliferative cell zone of the normal mucosa due to the horizontal cutting (HE, **e** x40; **f** x200).

**Supplementary Figure 5.**


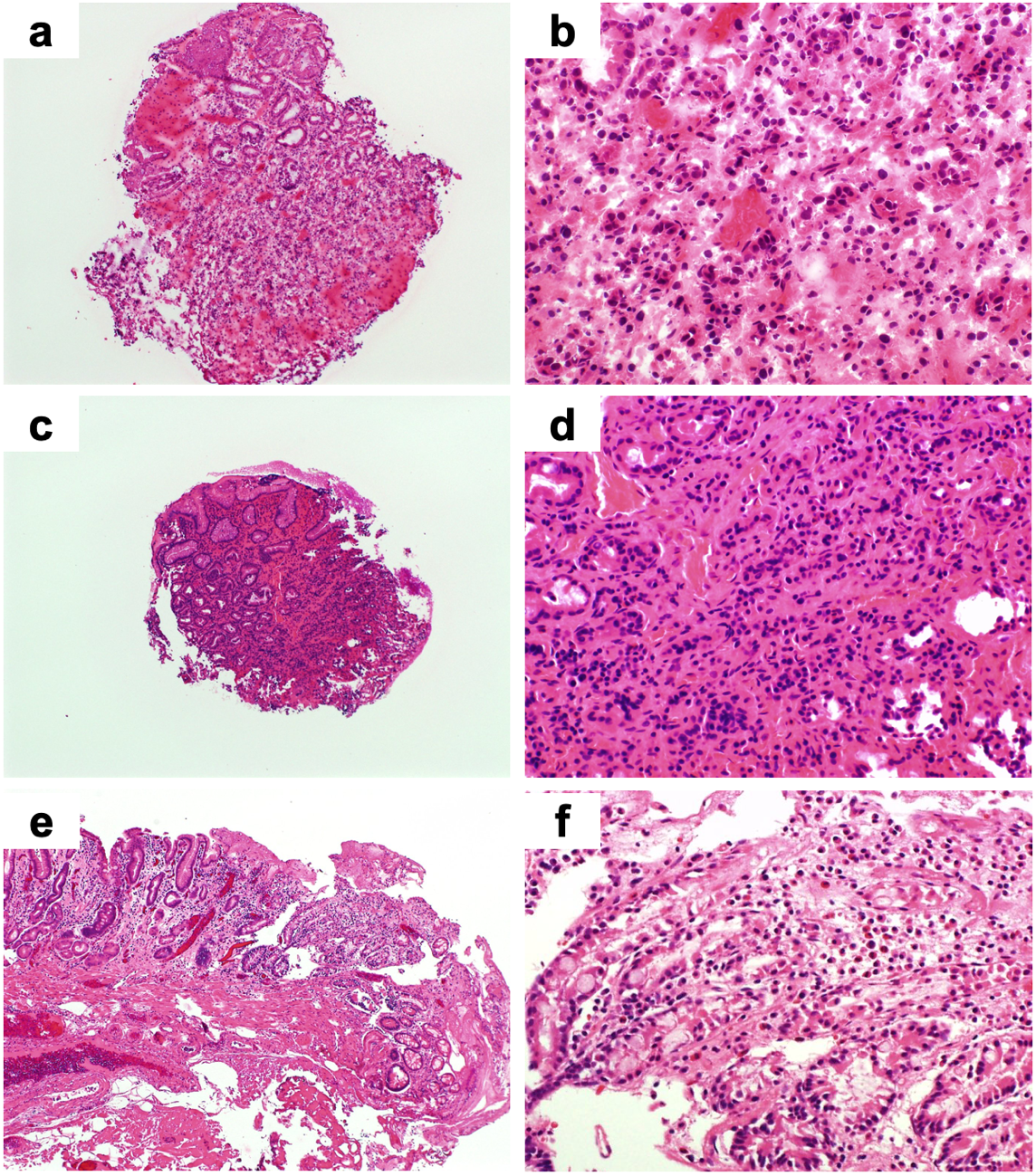


Pathological images of the non-cancerous frozen section diagnosed as “indefinite for neoplasia” (Case #8 in Table 4).

**a-b** The frozen section shows an amorphous appearance of the epithelium and connective tissue with irregularly shaped nuclei, such as spindle or circular, in the lower half of the image (HE, **a** x40; **b** x200). **c-d** The fixed biopsy specimen shows the similar findings as the frozen section (HE, **c** x40; **d** x200). **e-f** The ESD specimen shows fundic gland mucosa with severe atrophy and moderate intestinal metaplasia. In addition, thermal damage due to marking dots by coagulation are observed on the right side of the image. It is difficult to judge the changes in the epithelium and connective tissue due to thermal damage of the non-cancerous mucosa without information regarding coagulation during ESD (HE, **e** x40; **f** x200).
